# Genotoxic Effects in Swimmers Exposed to Disinfection By-products in Indoor Swimming Pools

**DOI:** 10.1289/ehp.1001959

**Published:** 2010-09-12

**Authors:** Manolis Kogevinas, Cristina M. Villanueva, Laia Font-Ribera, Danae Liviac, Mariona Bustamante, Felicidad Espinoza, Mark J. Nieuwenhuijsen, Aina Espinosa, Pilar Fernandez, David M. DeMarini, Joan O. Grimalt, Tamara Grummt, Ricard Marcos

**Affiliations:** 1 Centre for Research in Environmental Epidemiology, Barcelona, Spain; 2 Municipal Institute of Medical Research, Hospital del Mar, Barcelona, Spain; 3 CIBER Epidemiologia y Salud Pública, Barcelona, Spain; 4 National School of Public Health, Athens, Greece; 5 Grup de Mutagénesi, Departament de Genética i de Microbiologia, Universitat Autònoma de Barcelona, Bellaterra, Cerdanyola del Vallés, Spain; 6 Centre for Genomic Regulation, Barcelona, Spain; 7 Institute of Environmental Assessment and Water Research, Barcelona, Spain; 8 National Health and Environmental Effects Research Laboratory, U.S. Environmental Protection Agency, Research Triangle Park, North Carolina, USA; 9 Federal Environmental Agency, Bad Elster, Germany

**Keywords:** cancer, chlorination, disinfection by-products, genetics, genotoxicity, mutagenicity, swimming pools, water

## Abstract

**Background:**

Exposure to disinfection by-products (DBPs) in drinking water has been associated with cancer risk. A recent study (Villanueva et al. 2007; Am J Epidemiol 165:148–156) found an increased bladder cancer risk among subjects attending swimming pools relative to those not attending.

**Objectives:**

We evaluated adults who swam in chlorinated pools to determine whether exposure to DBPs in pool water is associated with biomarkers of genotoxicity.

**Methods:**

We collected blood, urine, and exhaled air samples from 49 nonsmoking adult volunteers before and after they swam for 40 min in an indoor chlorinated pool. We estimated associations between the concentrations of four trihalomethanes (THMs) in exhaled breath and changes in micronuclei (MN) and DNA damage (comet assay) in peripheral blood lymphocytes before and 1 hr after swimming; urine mutagenicity (Ames assay) before and 2 hr after swimming; and MN in exfoliated urothelial cells before and 2 weeks after swimming. We also estimated associations and interactions with polymorphisms in genes related to DNA repair or to DBP metabolism.

**Results:**

After swimming, the total concentration of the four THMs in exhaled breath was seven times higher than before swimming. The change in the frequency of micronucleated lymphocytes after swimming increased in association with higher exhaled concentrations of the brominated THMs (*p* = 0.03 for bromodichloromethane, *p* = 0.05 for chlorodibromomethane, *p* = 0.01 for bromoform) but not chloroform. Swimming was not associated with DNA damage detectable by the comet assay. Urine mutagenicity increased significantly after swimming, in association with the higher concentration of exhaled bromoform (*p* = 0.004). We found no significant associations with changes in micronucleated urothelial cells.

**Conclusions:**

Our findings support potential genotoxic effects of exposure to DBPs from swimming pools. The positive health effects gained by swimming could be increased by reducing the potential health risks of pool water.

Swimming in pools is an important recreational activity for hundreds of millions of people worldwide and has been associated with significant positive health benefits (Zwiener et al. 2007). Hygiene and water quality, especially infections caused by feces-associated microbes and protozoa, have been a priority for regulators and researchers (World Health Organization 2006). However, concerns have been raised regarding potential adverse health effects resulting from exposure to chemically disinfected swimming pool water (Zwiener et al. 2007).

As with drinking water, chlorination is the most common method of disinfection for swimming pools. The addition of chlorine to water results in the formation of hundreds of chlorination by-products because of the presence of organic matter (Richardson et al. 2007). Levels of disinfection by-products (DBPs) in swimming pool water are not necessarily higher than those in drinking water (Richardson et al. 2010). Swimming in an indoor pool, however, leads to a high uptake of compounds such as trihalomethanes (THMs), which are inhaled and absorbed by the skin (Whitaker et al. 2003; Xu and Weisel 2004, 2005). High levels of haloacetic acids also have been reported in swimming pools; however, these DBPs are likely not taken up at significant levels because they are nonvolatile, and uptake occurs mainly through ingestion. Another chemical class identified recently in chlorinated pools is nitrosamines (Walse and Mitch 2008), but their uptake via swimming has not been studied.

Epidemiological studies have shown that long-term consumption of chlorinated water and exposure to THMs at levels found currently in drinking water in many industrialized countries are associated with an increased risk of bladder cancer (Villanueva et al. 2004). A large study on bladder cancer in Spain was the first to examine exposure to THMs through ingestion of water and through inhalation and dermal absorption during showering, bathing, and swimming in pools (Villanueva et al. 2007). Participants with household THM levels > 49 μg/L had twice the risk of bladder cancer as those with levels < 8 μg/L. The risks associated with tasks resulting in high exposure via inhalation and dermal absorption were higher than those for ingestion. In the same study, an increased risk was found for subjects attending swimming pools [odds ratio = 1.6; 95% confidence interval (CI), 1.2–2.1]. In another study, Nelemans et al. (1994) hypothesized that DBPs could be partly responsible for increased risk for melanoma among swimmers.

Several DBPs, including some THMs, are genotoxic, and all four regulated THMs (chloroform, bromoform, bromodichloromethane, and chlorodibromomethane) are carcinogenic in rodents (reviewed by Richardson et al. 2007). Chloroform is not mutagenic; however, the brominated THMs are, and they are activated to mutagens by glutathione *S-*transferase theta-1 (GSTT1) (DeMarini et al. 1997; Pegram et al. 1997). Bromodichloromethane has been shown to induce mutagenic activity in human urine (Leavens et al. 2007). All regulated THMs other than chlorodibromomethane have been shown to induce DNA damage *in vitro* as detected by the comet assay, and some studies have found that chlorodibromomethane induced chromosomal aberrations and sister chromatid exchanges and that bromoform induced sister chromatid exchanges and micronuclei (MN) [International Agency for Research on Cancer (IARC) 1999; Richardson et al. 2007]. Extensive quantitative testing of the mutagenic and genotoxic potency of DBPs has shown that iodinated compounds such as dichloroiodomethane are generally more toxic than are brominated DBPs, and DBPs that are both iodinated and brominated are more genotoxic than are chlorinated DBPs (Richardson et al. 2007).

Metabolism of DBPs is mediated by enzymes from the glutathionine *S*-transferase (GST) and cytochrome P450 (CYP) families. Evaluation of polymorphisms in *GSTT1*, a gene involved in the metabolism of brominated THMs, has indicated significantly stronger associations between THM exposure and bladder cancer among subjects with functioning *GSTT1* (+/+ or +/− genotypes) than among subjects with deletions in both alleles (−/−) (Cantor et al. 2010). This was consistent with early studies showing that GSTT1 activated the brominated THMs, but not chloroform, to mutagens in a transgenic strain of *Salmonella* (DeMarini et al. 1997; Pegram et al. 1997). GSTZ1 catalyzes the oxygenation of dichloroacetic acid to glyoxylic acid and plays a critical role in the tyrosine degradation pathway and in alpha-haloacid metabolism (Board and Anders 2005). *CYP2E1*, *CYP1A2*, *CYP3A4*, and *CYP2A6* are involved in the metabolism of chloroform and bromodichloromethane (Allis and Zhao 2002; Gemma et al. 2003; Leavens et al. 2007; Zhao and Allis 2002), and *CYP2D6* variants have been found to modify THM blood levels after showering (Backer et al. 2008). Metabolic activation of bromodichloromethane by GSTT1 may result in the formation of 8-oxoguanidine DNA adducts that are repaired by base-excision repair through the expression of genes such as *OGG1* (8-oxoguanine DNA glycosylase), *APEX1* [APEX nuclease (multifunctional DNA repair enzyme) 1], and *XRCC1* (X-ray repair complementing defective repair in Chinese hamster cells 1). There is some evidence that genetic variants of *XRCC1* influence MN formation, as might variants in the *ERCC2* (excision repair cross-complementing rodent repair deficiency, complementation group 2) gene, which is part of the nucleotide-excision repair (NER) pathway (Iarmarcovai et al. 2008). On the other hand, chloroform exposure results in increased expression of *APEX1* in rat liver (Zidek et al. 2007). Polymorphisms in these DNA repair genes also have been associated with bladder cancer, which has been associated most consistently with DBP exposure.

Genotoxicity evaluations are used extensively in studies of health effects of environmental exposures. These assays can be carried out using easily obtainable human cells, such as peripheral blood lymphocytes (PBLs) and exfoliated urothelial cells from urine, and have been used to evaluate the genotoxicity of DBPs (Liviac et al. 2009; Ranmuthugala et al. 2003; Richardson et al. 2007). Methods used to detect primary DNA damage include the comet assay, which detects single- and double-strand breaks, alkali-labile sites, and transient DNA repair breaks (Dusinska and Collins 2008). Primary DNA damage can be repaired easily; thus, it is necessary to also use biomarkers of fixed damage, which are probably more relevant for human risk assessment. In this context, the MN assay is a well-validated methodology that provides a measure of both chromosome breakage and chromosome loss, and it has been shown to be a relevant biomarker for cancer risk (Bonassi et al. 2007).

Urinary mutagenicity has been examined in occupational and environmental settings for more than three decades as a general cost-efficient assessment of systemic genotoxicity (Cerná and Pastorková 2002). Although several experimental studies have evaluated the mutagenicity of DBPs, only Leavens et al. (2007) have evaluated the effect of a DBP on urinary mutagenicity in humans with controlled dermal and oral exposures; they found that urinary mutagenicity levels increased after exposure to bromodichloromethane, particularly among subjects exposed percutaneously compared with orally.

To evaluate the genotoxicity of swimming pool water in swimmers, we examined the above-mentioned biomarkers of genotoxicity in an experimental study in which adults swam for 40 min in a chlorinated, indoor swimming pool. We compared the biomarker results with an internal measure of exposure: the concentrations of four THMs in exhaled breath as determined in a companion study (Font-Ribera et al. 2010). We also evaluated the impact of genotype on the biomarker responses relative to THM exposure.

## Materials and Methods

### Study design

**Figure 1:**
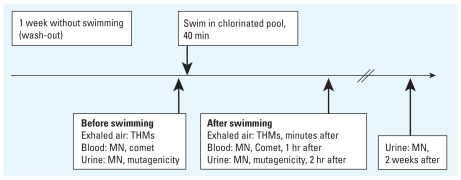
Design of the swimming pool study. Samples were collected before swimming and minutes (exhaled breath), 1 hr (blood for MN and comet), 2 hr (urine for mutagenicity testing), and 2 weeks (urine for MN in exfoliated cells) after swimming.

Fifty nonsmoking volunteers 18–50 years of age were recruited through open advertisements on the Internet and at local universities, avoiding any direct personal contact (e.g., e-mailing) because this is prohibited in research centers in Spain. A screening questionnaire was used to verify eligibility. Compensation was provided to subjects, who signed an informed consent form that acknowledged this compensation. We selected a single, indoor, 25-m-long chlorinated swimming pool in Barcelona, Spain, for the study, which was conducted during May, June, September, and October 2007. Figure 1 shows the design of the swimming pool study.

We asked subjects not to swim for 1 week before the swimming experiment. For the experiment, subjects were asked to swim for 40 min in the pool, and the time and distance of swimming were recorded for each individual. We selected 40 min based on our estimate of the usual time that noncompetitive swimmers swim. Biological samples (exhaled breath, blood, and urine) were collected both before and after swimming at specified time periods (Font-Ribera et al. 2010), and questionnaires were completed before swimming. THMs in exhaled breath were measured within a few minutes after swimming and before taking a shower. Blood samples were collected on average 1 hr after swimming, and urine samples were collected on average 2 hr after swimming. A second urine sample was collected 2 weeks after swimming; this sample was used for analysis of MN in exfoliated urothelial cells. One subject failed to adequately complete the procedures for the measurement of THMs in exhaled breath and was excluded, leaving 49 subjects for the final analysis. All laboratory analyses were blind concerning before or after swimming. The study protocol was approved by the ethics committee of the research center, and, as noted above, all subjects signed informed consent forms.

### Questionnaire

Subjects completed a self-administered questionnaire that included information on sociodemographics (e.g., age, education, occupation, basic residential and commuting information), detailed water-related habits (e.g., fluid ingestion, showers, baths, swimming pools), physical activity, medical history and drugs, and lifestyle (e.g., past smoking, second-hand smoke, use of hair dyes), as well as a food-frequency questionnaire validated in the Spanish population and a short 24-hr activity and recent-disease questionnaire. Measurement of physical activity during swimming was done indirectly by recording each individual’s swimming pattern (number of laps).

### Exposure assessment

A detailed description of measurements of THMs in air and exhaled breath before and after swimming is reported by Font-Ribera et al. (2010), and information on levels of DBPs in the water and water mutagenicity is reported by Richardson et al. (2010). THMs in air and water were determined at each swimming session following a specific protocol. There are distinct advantages of using exhaled breath to assess THM intake: It is noninvasive and provides a representative estimate of the concentration of contaminants in blood due to the gas exchange in the blood/breath interface in the lungs. Analysis of air and exhaled breath involved solid-phase adsorption on Tenax TA (Supelco, Bellefonte, PA, USA). Exhaled breath samples were collected using a portable system that consisted of a Haldane-Priestley tube modified to concentrate aliquots of exhaled breath from one or more exhalations. Water samples were analyzed with a purge-and-trap concentrator equipped with a Tenax silica gel-charcoal trap (Supelco).

### Comet analysis in PBLs

The comet assay was performed as described previously (Singh et al. 1988) with minor modifications. Blood samples were collected in Vacutainers containing EDTA. Samples were kept chilled, and the length of time between blood collection and sample processing was a few hours (Anderson et al. 1997). One hundred cells selected randomly (50 cells from each of the two replicate slides) were analyzed per sample. Olive tail moment (OTM) and percentage of DNA in the tail, used as measures of DNA damage, were computed using Komet software, version 5.5 (Kinetic Imaging, Liverpool, UK); [see also Supplemental Material (doi:10.1289/ehp.1001959)].

### MN analysis in PBLs

Blood was obtained from each subject by venipuncture using heparinized Vacutainers and sent immediately to the laboratory for the lymphocyte cultures. To determine the frequency of binucleated cells with MN and the total number of MN, a total of 1,000 binucleated cells with well-preserved cytoplasm (500/replicate) were scored for each subject. In addition, we scored 500 lymphocytes to evaluate the percentage of cells with one to four nuclei and calculated the cytokinesis block proliferation index (Surrallés et al. 1995). Microscopic scoring was performed on coded slides [see also Supplemental Material (doi:10.1289/ehp.1001959)].

### MN analysis in urothelial cells

Urine samples (~ 50 mL) were collected into plastic vials before swimming and again 2 weeks later; samples were then sent to the laboratory and processed the same day. We selected 2 weeks because this is the amount of time required for exfoliation of cells from the urothelium (Espinoza et al. 2008). We followed the criteria for MN evaluation suggested by Stick et al. (1983) as updated by subsequent guidelines by Fenech et al. (2003). The frequency of urothelial cells with MN and the total number of MN were determined for each analyzed subject. We scored only cells with a typical morphology corresponding to urothelial cells in order to avoid any kind of bias, especially in women where many squamous cells not of urothelial origin are observed. Although bacteria were present in a few urine samples, they did not interfere with the scoring [see also Supplemental Material (doi:10.1289/ehp.1001959)].

### Urine mutagenicity

Urine samples (30 mL) collected before and 90–120 min after exposure were evaluated for mutagenicity in the *Salmonella* (Ames) mutagenicity plate-incorporation assay (Maron and Ames 1983) in strain YG1024 with S9 mix. YG1024 is a frameshift strain derived from TA98 (*hisD3052*, Δ*uvrB*, *rfa*, pKM101) that contains acetyltransferase activity (Watanabe et al. 1990), and it has been used extensively for urinary mutagenicity studies (Cerná and Pastorková 2002). We used only one strain because of the limited availability of sample. The mutagenic potencies of samples, expressed as revertants per milliliter-equivalent (rev/mL-eq), were calculated from the slope of the regression over the linear portion of the dose–response curves. Slopes were calculated for 43 subjects with samples from before and after swimming that were sufficient for analyses of mutagenicity at three or more different concentrations [see also Supplemental Material (doi:10.1289/ehp.1001959)].

### Gene selection and genotyping

We examined genetic variants, including single-nucleotide polymorphisms (SNPs) and copy-number variants (CNVs), in three genes involved in the metabolism of DBPs (*GSTT1*, *CYP2E1*, and *GSTZ1*), four additional genes that may play a minor role in the metabolism of DBPs (*GSTT2B*, *GSTM1*, *CYP1A2*, and *CYP2D6*), and four DNA repair genes that could be relevant when examining results, particularly for the comet assay (*APEX1*, *ERCC2*, *OGG1*, *XRCC1*). We selected tag SNPs combined with the functional variants most likely to influence gene expression or function. In total, 3 CNVs and 17 SNPs were genotyped. For a complete list, see Supplemental Material, Tables 1–4 (doi:10.1289/ehp.1001959).

We performed SNP genotyping using the Sequenom platform (Sequenom, Inc., San Diego, CA). Two individuals with low genotyping frequency (< 75%) and two non-Caucasians were excluded from the genetic analyes. Genotyping failed completely for the following SNPs: rs11101815 and rs915908 in *CYP2E1*, rs1799793 in *ERCC2*, and rs28903081 in the *XRCC3* gene. The remaining 13 SNPs had a call frequency > 90% [see also Supplemental Material (doi:10.1289/ehp.1001959)].

### Statistical analysis

Paired *t*-tests were used to examine changes in the level of biomarkers before and after swimming. Associations between exposure to THMs measured in exhaled breath after swimming and changes in markers of genotoxicity before and after swimming were evaluated using linear regression. All analyses were adjusted for age and sex. We evaluated several other variables as potential confounders, including water consumption, source of water, antioxidant intake from diet, number of laps swum during the experiment (an indication of physical activity), and leisure-time physical activity. However, because estimated effects were modified only marginally by the inclusion of these variables in the models, we report results adjusted for age and sex only. To estimate the amount of variance in an end point due to the concentration of THMs in exhaled breath, we used unadjusted models to calculate the *r*^2^ values. The default *p*-value used to determine statistical significance was < 0.05.

We also tested for genotype deviations from Hardy-Weinberg equilibrium (Wigginton et al. 2005). Analysis of single-marker effect was performed assuming both a dominant and an additive (data not shown) genetic model, considering the most frequent allele as a reference category, and using logistic regression implemented in SNPassoc (version 1.5-1) from R statistical software, version 2.6.1 (R Development Core Team 2007). To evaluate interactions between changes in THMs and gene variants, we included an interaction term for dichotomous genotype and THMs in the linear regression models.

## Results

Among the 50 subjects who participated in at least part of the study, 66% were women, 96% were Caucasian, most were highly educated, and by selection criteria, all were nonsmokers, with approximately one-third being ex-smokers (Table 1). Sixty-eight percent of subjects were exposed regularly to second-hand smoke. About half played regular sports (once per week), and 11 (22%) swam at least once per month. The free chlorine level in the pool water (mean ± SD) was 1.17 ± 0.4 mg/L and the total THM level was 45.4 ± 7.3 μg/L. In pool air, the total THM level was 74.1 ± 23.7 μg/m^3^. We excluded one subject who failed to adequately complete the procedures for the measurement of THMs in exhaled breath, leaving 49 subjects for the final analysis. In exhaled breath, the THM levels after swimming were, on average, about 7 times those for before swimming. The average total THM levels before and after swimming were 1.2 and 7.9 μ/m^3^, respectively, and the corresponding average levels for the individual THMs were 0.7 and 4.5 μg/m^3^ for chloroform, 0.26 and 1.78 μg/m^3^ for bromodichloromethane, 0.13 and 1.2 μg/m^3^ for chlorodibromomethane, and 0.1 and 0.5 μg/m^3^ for bromoform (tribromomethane).

The average number of MN-positive cells per 1,000 binucleated lymphocytes increased nonsignificantly from 3.4 before swimming to 4.0 after swimming (Table 2). Likewise, the average frequency of MN in urothelial cells and the level of urinary mutagenicity also increased nonsignificantly after swimming relative to before swimming. In contrast, we observed a small but statistically significant decrease in the average amount of DNA damage in PBLs measured through the comet assay after swimming relative to before swimming (Table 2).

In the multivariate analysis, the change in the frequency of MN in PBLs before and after swimming was associated with the combined concentration of all four THMs measured in exhaled breath. Specifically, a 1-μg/m^3^ increase in total THMs in exhaled breath after swimming was associated with an average increase of 0.296 MN/1,000 cells; however, this increase was not significant (*p* = 0.09) (Table 3). We observed the largest increases in MN for the brominated THMs (Table 3), with statistically significant increases associated with exposure to bromodichloromethane (1.9 MN/1,000 cells; 95% CI, 0.21–3.63) and bromoform (5.04 MN/1,000 cells; 95% CI, 1.23–8.84). Based on the *r*^2^, the fraction of the variance in changes in MN frequency that was explained by exposure to bromodichloromethane was 10%; for bromoform this was 13%. Adjustment for potential confounders, including the number of laps swum during the experiment (as a measure of physical activity), resulted in only minor changes in effect estimates compared with those adjusted for age and sex only [see Supplemental Material, Figure 1 (doi:10.1289/ehp.1001959)].

Total or individual THM concentrations in exhaled breath were not associated with the level of DNA damage in PBLs as assessed by the comet assay, regardless of whether damage was quantified based on the OTM (Table 3) or the percentage of DNA in the tail (data not shown).

MN frequency in exfoliated urothelial cells was increased with a 1-μg/m^3^ increase in chlorodibromomethane (2.4 MN/2,000 cells; 95% CI, −3.3 to 8.2) and bromoform (4.3 MN/2,000 cells; 95% CI, −6.9 to 15.5), but estimates were not statistically significant (Table 3).

We observed an increase in urinary mutagenicity, measured as an increase in the slope of the dose–response curve (mutagenic potency) before and approximately 1.5 hr after swimming, for the combined concentration of the four THMs in exhaled breath as well as for the concentration of each individual THM (Table 3). However, these increases were statistically significant only for bromoform (5.27; 95% CI, 1.80–8.75; *p* = 0.004). Based on the *r*^2^, the fraction of the variance in change in urinary mutagenicity that was explained by exposure to bromoform was 16%.

We modeled interactions between dichotomous genotypes and changes in THMs after swimming to evaluate modification of estimated effects of THMs on measured outcomes by genetic variation of genes involved in the metabolism of DBPs or in DNA repair. Table 4 shows results of models to assess interactions between bromoform (the THM showing the most significant associations with the effect biomarkers) and polymorphisms in *GSTT1*, *GSTZ1*, and *CYP2E1* metabolism genes. Complete results for all gene variants examined are reported in Supplemental Material, Tables 1–4 (doi:10.1289/ehp.1001959).

Subjects with the null *GSTT1* genotype (−/−; a deletion in both copies of the gene) had lower frequencies of MN in urothelial cells and lower urinary mutagenicity than did those with one (+/−) or none (+/+) of the copies deleted, but differences were not statistically significant (Table 4). We did not observe a statistically significant modification by *GSTT1* of effects of THMs on MN in PBLs, although −/− individuals tended to have higher MN levels in lymphocytes than did −/+ and +/+ individuals. Statistically significant interactions were found between exposure to bromoform and *GSTZ1* (rs3177427) for MN in PBLs [β coefficient, 10.2 (95% CI, 3.0–17.3) and 1.4 (95% CI, −3.7 to 6.5) for GG vs. AG or AA genotypes, respectively] and between bromoform and *CYP2E1* (rs915906) for MN in urine [11.6 (95% CI, 2.5–20.8) and −23.2 (95% CI, −52.4 to 6.1) for TT vs. CT or CC genotypes, respectively; Table 4]. We also found statistically significant interactions between bromoform exposure and gene variants on MN in PBLs for *GSTT2B* [9.6 (95% CI, −0.11 to 19.3) and 1.95 (95% CI, −2.32 to 5.51) for +/+ vs. −/+ or −/− genotypes, respectively] and *APEX1* [9.5 (95% CI, 3.3–15.6) and −2.5 (95% CI, −9.4 to 4.5) for TT vs. GT or GG genotypes, respectively; see Supplemental Material, Table 1 (doi:10.1289/ehp.1001959)] and between bromoform and *GSTM1* on MN in urine [−23.1 (95% CI, −50.5 to 4.5) and 8.1 (95% CI, −2.1 to 18.3) for −/− vs. −/+ or +/+ genotypes, respectively; see Supplemental Material, Table 2]. No statistically significant interactions were found between bromoform and any of the gene variants associated with DNA damage that we assessed using the comet assay (Table 4; see also Supplemental Material, Table 4).

## Discussion

This is the first study of DBP genotoxicity among people who swam in a chlorinated pool. Biomarkers of genotoxic effects have been used extensively to evaluate potential health effects of environmental exposures, and the MN assay has been shown to be a predictive biomarker of cancer risk within a population of healthy subjects (Bonassi et al. 2007). In the present study, we observed increases in two biomarkers of genotoxicity (MN in PBLs and urinary mutagenicity) based on the change in brominated THM concentration in exhaled breath after swimming; however, there was no association with exposure to chloroform, which is not genotoxic (Richardson et al. 2007). We found no association between exposure to THMs in the pool and DNA damage in PBLs as measured by the comet assay. Associations were not dependent on confounding factors. We found some indication that responses to THMs were modified by variation in genes that metabolize these compounds, but there was limited power to evaluate gene–environment interactions.

The four THMs we evaluated are the most common DBPs in swimming pool water (Richardson et al. 2010). Although THMs are not considered to be the most toxic of the DBPs, all four are carcinogenic in rodents (Richardson et al. 2007). The brominated THMs have been shown to be mutagenic after activation by *GSTT1*, and some of them have been shown to induce chromosomal aberrations, sister chromatid exchanges, and/or MN in animal and human cells (IARC 1999; Richardson et al. 2007). In contrast, chloroform is not genotoxic (Richardson et al. 2007), and unlike the brominated THMs, it is not activated by *GSTT1* (Pegram et al. 1997). Thus, our finding of an association between exposure to brominated THMs and an increased response among various genotoxicity biomarkers—but the absence of such an association with chloroform exposure—is consistent with the toxicology of these THMs.

Our results are also consistent with extensive quantitative genotoxicity data on DBPs showing that brominated DBPs are generally more genotoxic and carcinogenic than chlorinated DBPs (Plewa et al. 2008; Richardson et al. 2007). In our study, we evaluated only THMs that are known to be the most common DBPs in swimming pool water and that exhibit high uptake by swimmers (Zwiener et al. 2007). Levels of haloacetic acids can also be high in swimming pools; however, the uptake of these DBPs may be low because the haloacetic acids are not volatile and are not adsorbed efficiently by the skin (Xu et al. 2002). Among the chemical classes of DBPs, the rank order of the combined cytotoxicity and genotoxicity in Chinese hamster ovary cells was halonitromethanes > haloacetamides > haloacetonitriles > haloacetic acids > halomethanes (Richardson et al. 2007). Future studies of swimmers should evaluate more completely the uptake and potential effects of a range of DBPs and other compounds present in pool water.

We found tthat evaluation of modification of environmental exposure by genetic polymorphisms was of low statistical power given the relatively small sample size; thus, these findings should be interpreted with caution. The main hypotheses focused on a potential modification of the effect by variants in a few genes (*GSTT1*, *GSTZ1*, and *CYP2E1*) that code enzymes that are important for DBP metabolism (Richardson et al. 2007). Similar to what has been shown for mutagenesis in bacteria and DNA adducts in rodents (DeMarini et al. 1997; Pegram et al. 1997; Ross and Pegram 2003), individuals with the *GSTT1* null genotype had lower frequencies of MN in urothelial cells and lower urinary mutagenicity than those with at least one functional allele; however, in our study, the differences were not statistically significant. We did not observe modification by *GSTT1* of effects of THMs on MN in PBLs, consistent with a lack of *GSTT1* expression in lymphocytes (Wang et al. 2000). In contrast, individuals bearing *GSTT2B* +/+ had higher numbers of MN in PBLs than did other subjects. The CNV encompassing *GSTT2B*, which modifies *GSTT2* gene expression, is in linkage disequilibrium with the *GSTT1* CNV (Zhao et al. 2009). All three of these genes are located in the same cluster, and combined effects cannot be excluded; however, the role of *GSTT2* and *GSTT2B* genes on DBP detoxification is unknown. Limited experimental data are available for *GSTZ1* and *CYP2E1* in relation to DBP exposure. In the present study, we identified differences between subjects of different genotypes, with those associated with *CYP2E1* being statistically significant. These findings should be verified in further studies.

Potential confounding was minimized in our study, which involved comparing individuals with themselves before and after an exposure over a limited time period. Any changes in biomarkers were likely attributed to one of three factors: exposures related to swimming pools, swimming itself, or chance. As expected, control of other lifestyle factors and environmental exposures did not modify results. Some studies (Schiffl et al. 1997) have shown physical activity to be associated with genotoxicity through an effect on oxidative stress, but the results are not consistent (Battershill et al. 2008; Stefanie et al. 2008). In our study population, adjustment for the intensity of physical activity during swimming indicated no confounding of effect estimates for exposure to DBPs.

Confounding could be more of a problem for the analysis of MN in exfoliated urothelial cells, which were collected 2 weeks after swimming. Although we did control for several lifestyle factors in the analysis, it is still possible that results in urine could have been affected more by uncontrolled confounding. Chance could be an explanation for some of the results and is particularly a problem for the evaluation of gene–environment interactions. In the main analyses of exposure and effect biomarkers, however, we made only a few comparisons, and the issue of chance findings due to multiple comparisons was minor. In addition, the identification of more pronounced genotoxic effects for the potentially more toxic brominated compounds compared with chloroform argues against an effect of chance.

The timing of the collection of biological samples is crucial when evaluating biomarkers of effect. Because of constraints in the study protocol, the first samples were collected during the 2-hr period after swimming—and 2 weeks after swimming for evaluation of exfoliated urothelial cells. Because of the lack of previous studies of this type with swimmers, we had no precedence to follow, and the timing of blood collection that we used might not have been the most appropriate for some of the assays. Specifically, it is possible that collecting blood approximately 1 hr after swimming for chemicals that are metabolized rapidly and that are of relatively low toxicity might not necessarily be appropriate for the comet assay because DNA damage induced by DBPs may already have been repaired before the sample is collected (Komaki et al. 2009; Liviac et al. 2009). However, studies in rodents frequently assess DNA damage in lymphocytes 3–4 hr after exposure. We collected urine 2 hr after swimming for mutagenicity analysis and also 2 weeks after swimming for MN analysis. This 2-week time period was selected to allow time for the exfoliation of cells from the urothelium exposed at the time of the experiment (Espinoza et al. 2008).

Swimming has significant positive health effects related to the benefits of exercise and has some advantages over land-based activities for people of all ages and physical abilities (Zwiener et al. 2007). To retain the positive aspects of aquatic activities, regulators and researchers have turned their attention to the hygienic aspects of the quality of pool water, as well as of its chemical composition. It will be important to maintain microbial disinfection while minimizing potentially harmful DBPs. The goal is to maintain the positive health effects of swimming while reducing other potential adverse health risks.

## Conclusion

We found that exposure to brominated THMs through swimming in chlorinated pools was associated with increases in genotoxicity biomarkers. When we used different genotoxicity and mutagenicity assays, our findings were consistent. We found that only brominated THMs were associated with higher genotoxicity; chloroform was not. Our results are also consistent with the presence of mutagenic and genotoxic DBPs in pool water and the mutagenic activity of the pool water (~ 1,200 rev/L-eq in *Salmonella* strain TA100), of which, as noted by Richardson et al. (2010), were present at levels similar to those found in drinking water. However, Richardson et al. (2010) found that the concentrations of nitrogen-containing DBPs were higher in pool water than in drinking water. Although our study had low power to estimate unambiguously the effects of genetic variation on responses to chemical exposures during swimming, it appears plausible that such variation exists. Our findings, which should be verified in larger studies, indicate that the positive health effects gained by swimming could be increased by reducing the potential heath risks of pool water.

## Figures and Tables

**Table 1:** Characteristics of the study population.

Characteristic	Number (%)
Sex	
Male	17 (34)
Female	33 (66)

Age (years)	
Mean ± SD	30.1 ± 6.1
Range	20–51

Ethnicity	
Caucasian	48 (96)
Other	2 (4)

Education^a^	
Secondary	4 (8)
University	45 (92)

Tobacco	
Never smokers	36 (72)
Ex-smokers	14 (28)

Second-hand smoke	
Yes	34 (68)
No	16 (32)

Regular swimming (≥ once/month)	
Yes	11 (22)
No	39 (78)

Regular sport (≥ once/week)	
Yes	27 (54)
No	23 (46)

a Missing value for one subject.

**Table 2:** Change in mean values of biomarkers before and after swimming.

	*n*		Mean ± SD		
Biomarker	Before	After	Before	After	*p*-Value^a^
MN-PBL	49	49	3.4 ± 2.4	4.0 ± 2.8	0.235
OTM-comet-PBL	49	49	1.5 ± 0.7	1.3 ± 0.6	0.008
MN-urothelial	33	33	9.0 ± 9.3	10.3 ± 7.4	0.350
Urine mutagenicity (rev/mL-eq)	43	43	0.6 ± 2.3	1.2 ± 2.2	0.257

Abbreviations: MN-PBL, MNs/1,000 binucleated cells; MN-urothelial, MNs per 2,000 cells; OTM-comet-PBL, OTM/100 cells.

a Paired *t*-test.

**Table 3:** Changes in MN frequency and OTM in PBLs, MN frequency in exfoliated urothelial cells, and urine mutagenicity with a 1-μg/m^3^ increase in total or individual THMs in exhaled breath.

Biomarker, exposure	β (95% CI)	*p*-Value
MN-PBL		
Total THMs	0.30 (−0.05 to 0.64)	0.09
CHCl_3_	0.29 (−0.27 to 0.85)	0.31
CHCl_2_Br	1.92 (0.21 to 3.63)	0.03
CHClBr_2_	1.71 (−0.02 to 3.44)	0.05
CHBr_3_	5.04 (1.23 to 8.84)	0.01

OTM-comet-PBL		
Total THMs	−0.02 (−0.07 to 0.04)	0.53
CHCl_3_	−0.02 (−0.10 to 0.06)	0.64
CHCl_2_Br	−0.04 (−0.30 to 0.23)	0.79
CHClBr_2_	−0.14 (−0.40 to 0.13)	0.30
CHBr_3_	−0.23 (−0.83 to 0.37)	0.45

MN-urothelial cells		
Total THMs	−0.10 (−1.19 to 1.01)	0.86
CHCl_3_	−0.47 (−2.11 to 1.18)	0.57
CHCl_2_Br	−0.46 (−6.50 to 5.58)	0.88
CHClBr_2_	2.44 (−3.32 to 8.20)	0.40
CHBr_3_	4.29 (−6.87 to 15.45)	0.44

Urine mutagenicity (rev/mL-eq)		
Total THMs	0.24 (−0.11 to 0.58)	0.17
CHCl_3_	0.33 (−0.22 to 0.89)	0.23
CHCl_2_Br	0.61 (−1.12 to 2.35)	0.48
CHClBr_2_	0.92 (−0.75 to 2.59)	0.27
CHBr_3_	5.27 (1.80 to 8.75)	0.004

Abbreviations: CHBr_3_, bromoform; CHCl_2_Br, bromodichloromethane; CHCl_3_, chloroform; CHClBr_2_, chlorodibromomethane; MN-PBL, MNs/1,000 binucleated cells; MN-urothelial, MNs/2,000 cells; OTM-comet-PBL, OTM/100 cells.

a β-Coefficients represent a change in the biomarker level for a 1-μg/m^3^ change in THMs in exhaled air measured after swimming.

**Table 4:** Interaction between polymorphisms in three genes involved in DBP metabolism and the effects of exposure to bromoform on MN and the comet assay in PBLs, MN in exfoliated urothelial cells, and urinary mutagenicity.

		MN in PBLs		MN in exfoliated urothelial cells		Urinary mutagenicity		Comet assay	
Gene	Genotype	*n*	β (95% CI)	*n*	β (95% CI)	*n*	β (95% CI)	*n*	β (95% CI)
*GSTT1*	−/−	16	7.8 (1.9 to 13.7)	10	−5.2 (−48.6 to 38.3)	14	1.9 (−4.2 to 8.0)	16	−0.04 (−0.8 to 0.7)

	−/+, +/+	30	3.4 (−1.6 to 8.3)	23	1.7 (−11.1 to 14.6)	26	7.6 (3.0 to 12.2)	30	−0.4 (−1.3 to 0.4)

*CYP2E1*									
rs2070673	TT	27	4.4 (−0.4 to 9.1)	21	10.6 (0.9 to 20.2)	22	6.9 (0.7 to 13.0)	27	−0.3 (−1.1 to 0.5)
	AT, AA	16	−0.03 (−7.5 to 7.5)	10	−11.5 (−41.1 to 18.1)	15	3.0 (−2.9 to 9.0)	16	0.05 (−1.3 to 1.4)
rs915906	TT	28	6.7 (2.2 to 11.1)	22	11.6 (2.5 to 20.8)*	22	5.8 (1.8 to 9.9)	28	−0.4 (−1.03 to 0.2)
	CT, CC	14	−5.9 (−14.1 to 2.3)	9	−23.2 (−52.4 to 6.1)*	14	3.1 (−4.0 to 10.2)	14	−0.1 (−2.2 to 2.0)
rs915907	CC	30	3.7 (−1.8 to 9.1)	22	12.1 (−1.3 to 25.5)	27	4.9 (−0.9 to 10.6)	30	0.4 (−0.5 to 1.3)
	CA, AA	14	6.6 (−2.0 to 15.1)	9	9.8 (−12.3 to 31.9)	11	5.4 (−5.1 to 15.8)	14	0.9 (−1.7 to −0.04)
rs2515641	CC	35	5.8 (1.7 to 9.9)	25	7.6 (−3.2 to 18.4)	29	5.5 (1.1 to 10.0)	35	−0.3 (−0.9 to 0.3)
	CT	9	−0.4 (−14.1 to 13.3)	6	−11.0 (−23.6 to 1.6)	9	2.9 (−9.7 to 15.5)	9	0.8 (−2.0 to 3.5)
rs2249695	CC	25	4.2 (−0.8 to 9.2)	21	10.6 (0.9 to 20.2)	21	7.3 (2.8 to 11.9)	25	−0.3 (−1.1 to 0.5)
	CT, TT	19	7.5 (1.1 to 13.8)	10	−11.5 (−41.1 to 18.1)	17	2.5 (−2.7 to 7.6)	19	−0.1 (−1.1 to 0.8)

*GSTZ1*									
rs3177427	GG	22	10.2 (3.0 to 17.3)*	19	−0.2 (−19.4 to 19.0)	21	3.5 (−1.8 to 8.7)	22	−0.2 (−1.2 to 0.7)
	AG, AA	22	1.4 (−3.7 to 6.5)*	12	8.7 (−5.6 to 22.9)	17	8.7 (2.0 to 15.4)	22	−0.5 (−1.5 to 0.5)
rs1046428	CC	29	3.3 (−1.0 to 7.5)	20	5.8 (−5.7 to 17.2)	24	6.5 (1.9 to 11.2)	29	−0.3 (−1.1 to 0.4)
	CT, TT	14	−5.4 (−18.5 to 7.8)	11	21.1 (−7.6 to 49.8)	13	−2.1 (−18.9 to 14.7)	14	0.6 (−1.9 to 3.2)

* *p* < 0.05.
